# Retraction: Utilization of solid mine waste in the building materials for 3D printing

**DOI:** 10.1371/journal.pone.0345488

**Published:** 2026-03-23

**Authors:** The PLOS One Editors

Following the publication of this article [1], concerns were raised about results presented in Fig 10. Specifically:

- Similarities were noted between multiple regions within both Figs 10a and 10b.
- Multiple areas in Figs 10a and 10b appear discontinuous with adjacent regions of the images.

In light of these concerns, which call into question the integrity and reliability of the published results, the *PLOS One* Editors retract this article,

All authors either did not respond directly or could not be reached.
